# Effects of exercise on cardiovascular performance in the elderly

**DOI:** 10.3389/fphys.2014.00051

**Published:** 2014-02-20

**Authors:** Carlo Vigorito, Francesco Giallauria

**Affiliations:** ^1^Department of Translational Medical Sciences, University of Naples Federico IINaples, Italy; ^2^School of Science and Technology, University of New EnglandArmidale, NSW, Australia

**Keywords:** exercise, elderly, cardiovascular system, adrenergic system, vascular diseases

## Abstract

Progressive aging induces several structural and functional alterations in the cardiovascular system, among whom particularly important are a reduced number of myocardial cells and increased interstitial collagen fibers, which result in impaired left ventricular diastolic function. Even in the absence of cardiovascular disease, aging is strongly associated to a age-related reduced maximal aerobic capacity. This is due to a variety of physiological changes both at central and at peripheral level. Physical activity (PA) appears in general to have a positive effect on several health outcomes in the elderly. This review aims to illustrate the beneficial effects of exercise on the physiologic decline of cardiovascular performance occurring with age. Furthermore, it will be stressed also the positive effect of physical activity in elderly patients affected by cardiovascular diseases, such as heart failure and hypertension, and multiple comorbidities which may significantly worse prognosis in this high risk population.

## Aging and exercise effects in healthy elderly

Aging induces several structural and functional alterations in the cardiovascular system (Lakatta and Levy, 2003). Among central factors, a reduced maximal heart rate (HR) response to exercise due to beta1 receptors down-regulation plays a major role (Brubaker and Kitzman, 2011); among peripheral factors, progressive aging is associated to peripheral muscle cell biochemical and functional changes including reduction of skeletal muscle cells, decrease of Myosin Heavy Chain (MHC) I and IIa muscle fibers and impaired muscle oxidative capacity (Konopka et al., 2011).

Physical activity (PA) appears in general to have a positive effect on several health outcomes in the elderly: several epidemiologic studies have shown that the increase in the level of physical activities is associated to improved health outcomes (Sattelmair et al., 2009).

Aerobic (endurance) exercise programs in healthy elderly can have multiple beneficial effects on several health outcome, including a reduction in the decline in cardiovascular performance associated with physiologic aging, an improvement in physical function (Hollmann et al., 2007).

Resistance or strength training is particularly effective in increasing muscle mass and strength and improving several measurements of physical performance (Mangione et al., 2010). Integration of balance and strength training into daily life activity appears particularly efficient in reducing the rate of falls in older people and disability, and in improving the overall quality of life (QoL) (Clemson et al., 2012).

Cardiovascular performance can be measured at submaximal or maximal performance level. The 6 min Walking Test is the more widely used method of estimating submaximal performance, and is very useful in the elderly patients with comorbidities or frailty who is unable to perform a maximal exercise stress test. Maximal exercise performance can be measured by exercise stress test with extrapolation of metabolic equivalents (MET) or O_2_ consumption, or by a cardiopulmonary exercise stress test, with direct measurements of O_2_ consumption and CO_2_ production, in addition to many other derived parameters (Cohen-Solal, 1996).

Several studies have shown that aerobic exercise training improves measurements of cardiovascular performance in healthy elderly as expressed by an increase in peak VO_2_ (Fujimoto et al., 2010) and an improvement of other parameters of functional and prognostic relevance such as the VE/VCO_2_ slope, the ventilatory aerobic threshold, and the Heart Rate Recovery (HRR), a parameter expressing the sympatho-vagal balance (Giallauria et al., 2005; Fujimoto et al., 2010).

The mechanisms underlying the aerobic exercise-induced increase in peak VO_2_ in the healthy elderly are multifactorial and possibly recognize an improvement in both central and peripheral mechanisms of adaptation to exercise.

At the central level, aerobic exercise improves the chronotropic incompetence of the elderly, secondary to beta1 receptor down regulation, leading to an improved HR response to exercise (Brubaker and Kitzman, 2011); on the other side, Fujimoto et al. (2010) have shown that 1 year of endurance exercise training in a very selected healthy elderly population leads to an increase in exercise cardiac output (CO) (and thus in peak VO_2_) due mainly to increase in stroke volume, without changes in peripheral artero-venous (A-V) O_2_ difference. In old healthy subjects, it has been demonstrated that physical training ameliorates age-related deterioration of cardiac function in terms of enhanced left ventricular inotropic response to catecholamines (Spina et al., 1998). Contrasting data have been reported by other authors who described unchanged left ventricular systolic performance (Stratton et al., 1992) in response to adrenergic stimulation after training in the elderly. However, it is important to underline that exercise training also enhances vagal tone (Levy et al., 1966), which could mask the favorable effect of exercise on cardiac β-adrenergic responsiveness. Thus, exercise training is able to curb the detrimental effects of sustained neurohumoral activation in HF patients, with positive effects on cardiac function and peripheral vasoconstriction, ultimately improving exercise tolerance.

It is still uncertain whether the age-associated impairment in diastolic function may be reduced by exercise training, since endurance exercise training had only minimal effects on Doppler measures of LV diastolic function in healthy elderly (Prasad et al., 2007; Fujimoto et al., 2010).

At the peripheral level, aerobic exercise may improve peak VO_2_ by several mechanisms, such as an increased perfusion to exercising muscles due to favorable changes in endothelial dysfunction (Desouza et al., 2000) and in arterial compliance (Fujimoto et al., 2010), or an increased O_2_ extraction by the working muscles. Previous studies reported that exercise training in elderly subjects indeed increased peak AV O_2_ difference (McGuire et al., 2001) but this finding was not observed in other studies (Fujimoto et al., 2010). These discrepancies may depend from the different age of the study subjects, since the effects of training on peripheral oxygen utilization declines with progressive aging and probably a more intense training is required to increase peripheral oxygen extraction in very elderly subjects.

Improved exercise-induced extraction at muscle level might be due to an aerobic training-induced favorable biochemical changes, as indicated by increase in Myosin Heavy Chain I (MHC I) protein and mRNA, an oxidative MHC I phenotype which is beneficial for a more efficient O_2_ utilization during exercise by skeletal muscle of older subjects, and that is directly correlated with improvements in whole muscle power (Rinaldi et al., 2006; Konopka et al., 2011). These favorable changes at muscle level translate in improved physical and cardiovascular performance in elderly subjects. Furthermore, also an exercise-induced improvement in myofiber contractile function (Harber et al., 2012) contributes to an improved O_2_ efficient utilization by the skeletal muscle in older individuals.

There are some important gender differences in these changes, since the increase in peak VO_2_ after endurance training in healthy elderly men appears primarily due to an augmented peak stroke volume and CO and to a lesser extent to increased A-VO_2_ Difference. In contrast, the exercise training- induced increased peak VO_2_ in healthy elderly women is due entirely to an increase in peak A-VO_2_, with no changes in stroke volume, HR, and CO after training (Spina et al., 1993).

Resistance training has been shown to significantly increase skeletal muscle mass and strength in healthy elderly men and women (Frontera et al., 1990; Haykowsky et al., 2005), with favorable effects on physical function and QoL, and with fewer changes in maximal cardiovascular performance.

## Effects of phyisical activity on age-related increase of oxidative stress in the cardiovascular system

Aging is characterized by altered regulation of many genes implicated in stress resistance and processes of tissue regeneration and repair. In particular, old animals are intrinsically less resistant to oxidative stress (Corbi et al., 2012a,b). Although physical activity can increase oxidative stress, it is also able to counterbalance the deleterious effects of reactive oxygen species (ROS) accumulation by activation of several antioxidant systems, such as Super Oxide Dismutases (SODs), Heat shock proteins (HSPs) and catalase (Corbi et al., 2012a,b), with consequent reduction of ROS. In part, it seems that the positive effects of the physical exercise on aging heart in terms of antioxidant activity could be ascribable to a greater expression and activity of SOD and HSPs. It has been shown that a physical training program induced high levels of SOD and increased HSP70 and of HSP27 expression in trained old rats compared to sedentary old and young rats (Rinaldi et al., 2006). Physical activity has been demonstrated able to reduce generation of oxidants during ischemia-reperfusion damage and to have a calcium-protective role via activation of the ROS scavenger MnSOD. This better oxidative status consequent to a correct program of physical activity is partially responsible for some benefits such as decreased arterial stiffness, improved endothelial function and metabolic and clotting setting, and reduced body weight.

## Exercise in sarcopenic and frail patients

Sarcopenia is a geriatric clinical syndrome very frequent in the elderly characterized by Low muscle mass, low muscle strength and low physical performance due to a combination of factors such as the progressive ageing, nutrition problems, sedentary habit, chronic diseases, such as diabetes (Marciano et al., 2012; Paolillo et al., 2013) or drugs (Cruz-Jentoft et al., 2010). Sarcopenia leads to disability, risk of falls and fractures, and death. Frailty is a geriatric syndrome characterized by an increasing vulnerability to adverse health outcomes resulting from age-related cumulative declines across multiple physiologic systems, and leading to falls, hospitalization, institutionalization and death (Fried et al., 2001; Rengo et al., 2012d). Many patients with sarcopenia are also frail, but frailty definition encompasses broader domains of general elderly health beside sarcopenia. In sarcopenic and frail patients, several types of exercise are able to improve the sarcopenia frequently present with very advanced age and often associated with a frailty status.

In sarcopenic or frail patients resistance exercise training (RET) is capable to improve lean tissue mass, muscle strength and several physical performance measures such as time up and go, stair-climbing power, aerobic capacity, gait speed and 6 min walking distance, which are all consequences of increased muscle mass or strength (Liu and Latham, 2009; Peterson et al., 2010). In the medium-long term, RET interventions also improve several other health related outcomes, such as perceived QoL, functional independence, risk of falls, gait balance, and depressive symptoms (Gillespie et al., 2009).

The same favorable effects of RET have been also demonstrated in frail elderly patients (Mühlberg and Sieber, 2004). Improvement in muscle strength appears proportional to the intensity of resistance exercise and is larger in sarcopenic elderly than in frail patients with comorbid conditions or with functional limitations (Mangione et al., 2010).

Endurance exercise, compared to strength exercise training, leads only to a minor increase in muscle mass or strength in sarcopenic or frail patients, but is usually associated to a more pronounced improvement in aerobic exercise capacity (Frankel et al., 2006). Therefore, an endurance training program should be added to a RET program in a sarcopenic or frail patient to reinforce the functional short and long term beneficial effects of RET.

## Exercise in elderly patients with heart failure and reduced LV ejection fraction (HFREF)

Many studies have described the exercise training-induced adaptation of cardiovascular performance in HFREF (the more classic HF, that has been most widely studied in this respect).

In HFREF patients, favorable changes in exercise capacity with aerobic exercise training have been observed in elderly patients. This is particularly important since, in subjects with heart failure, exercise capacity (Fleg et al., 2000), such as natriuretic peptides (Savarese et al., 2013) is an independent predictor of long-term survival, although the mechanisms involved in the exercise-induced reduction in mortality are unclear and probably multifactorial. The mechanisms underlying the favorable effects of aerobic exercise on cardiovascular capacity may consist in a combination of central mechanisms, such as an increased CO and favorable left ventricular remodeling and modulation of sympathetic nervous system overactivity by restoring Beta-adrenergic receptor signaling (Rengo et al., 2012a,b,c, 2014; Femminella et al., 2013; Salazar et al., 2013) and of peripheral mechanisms, such as an increase in skeletal muscle perfusion, oxygen extraction and utilization, favorable phenotypic changes in skeletal muscle cell and reduced ergoreflexes. Although the majority of the patient population included into these studies were elderly patient. they cannot be easily transposed to the real life, very elderly population with HFREF.

Severe exercise intolerance or fatigue, and dyspnea on exertion are the major symptomatic hallmark of HF. The most reliable and widely adopted method for objectively evaluate exercise intolerance in these patients is the measurement of peak O_2_ consumption (peak VO_2_) and other parameters obtained at a maximal cardiopulmonary exercise test. Several studies performed in HFREF patients (independently from age) have shown that endurance training induces a significant increase in peak VO_2_ of about 20–25%, which is correlated to survival and is one of the main prognostic indicators in HFREF (Pina et al., 2003). Peak VO_2_ may be calculated by the Fick formula to be the product of CO × A-VO_2_ Difference, while CO is the product of HR and stroke volume. Thus, each of these components may influence the exercise training induced increase in peak VO_2_.

Although widely studied, the precise mechanisms underlining the aerobic exercise-training induced increase in peak VO_2_ in HFREF patients is still controversial. Chronotropic incompetence plays a major role in reduced exercise intolerance in HFREF (Brubaker et al., 2006). Hambrecht et al. (2000a) reported an increased in peak exercise HR and CO, while other Authors did not confirm these findings (Sullivan et al., 1988; Jette et al., 1991; Belardinelli et al., 1996), reporting that rather an increased peak A-VO_2_ Difference was the major contributor to the endurance training-induced improved exercise capacity in these patients. Other authors (Dubach et al., 1997) on the other side reported an endurance training- increase in both peak exercise CO and A-VO_2_ Difference in HFREF patients. Recent experimental study has shown that ET, by modulation of post-receptor transcriptional factors, reverses myocardial beta-adrenergic receptor down-regulation, thus leading to an improved contractility reserve during exercise (Leosco et al., 2008; Cannavo et al., 2013a,b; Rengo et al., 2013). However, it is still uncertain whether these experimental data can translate into an increase in cardiac performance in the clinical setting, particularly in the elderly population with HFREF.

As far as exercise training induced changes in LV volumes, the results of prior mechanistic studies of endurance training in patients with HFREF indicate that endurance exercise training did not significantly enhance peak exercise left ventricular end diastolic or end-systolic volume in these patients, and rather lead to a favorable inverse LV remodeling or a reduced progression to LV remodeling, with a minor increase in resting LV contractility (Giannuzzi et al., 2003; Haykowsky et al., 2007; Giallauria et al., 2008, 2013; Van der Meer et al., 2012). In addition, ET improves endothelium-dependent coronary dilatation (Hambrecht et al., 2000b) and possibly myocardial perfusion. The demonstration of a favorable effect of ET on endothelial progenitor cells and vascular grow factors opens a new research avenue for understanding the central cardiac effects of exercise in patients with HFREF (Sarto et al., 2007). Whether an ET-induced improvement of ischemic preconditioning contributes to myocardial protection in patients with HFREF is still debated (Piepoli et al., 2010).

The factors contributing to the aerobic exercise-induced increased of A-VO_2_ Difference include increased muscle flow to exercising muscles, improved microcirculatory function, or improved O_2_ utilization by skeletal muscles. In HFREF patients, endothelium-dependent dilation of peripheral arterial vessels is impaired and improves after aerobic exercise training (Hornig et al., 1996), leading to an increased blood supply and improved distribution to exercising muscle.

However, also peripheral muscular adaptation play a major role in the aerobic exercise-induced increased of A-VO_2_ Difference; several cross-sectional studies have shown that HFREF patients present baseline alterations in skeletal muscle mass, composition, and function that correlate with their reduced exercise capacity (Sullivan et al., 1990; Wilson et al., 1993; Duscha et al., 1999). Several studies in HFREF patients have demonstrated that exercise training produces a variety of favorable skeletal muscle adaptations, including increased percent oxidative fibers, oxidative enzyme activity, and capillary density, leading to a more efficient muscle metabolism and O_2_ utilization (Stratton et al., 1994; Hambrecht et al., 1995, 1997; Magnusson et al., 1996; Tyni-Lenne et al., 1997). Gielen et al. (2012) have recently shown that that endurance exercise training reduces MuRF-1, a component of the ubiquitin-proteasome system involved in muscle wasting and sarcopenia often associated with HFREF, suggesting that exercise training blocks ubiquitin-proteasome system activation in both younger and older HFREF patients. The improvement in catabolic-anabolic imbalance shown in this study may contribute to an improved peripheral muscle function and cardiovascular performance in older CHF patients.

Among peripheral factors influencing the symptoms on exercise (particularly dyspnea on effort) in HFREF patients, an important role is played by an increased ventilator drive through reduced lactate production at submaximal exercise level and an enhanced muscle ergoreflexes secondary to skeletal muscle myopathy (Piepoli et al., 2010). Aerobic ET reduced ventilator drive as shown by the decrease of VE/VCO_2_ slope at cardiopulmonary stress test. In addition to endurance aerobic training, respiratory muscles training contributes to improved ventilation, and may reflect in an improved peripheral hemodynamics (Chiappa et al., 2008).

Taken together, studies performed in HFREF patients suggest that the rise in peak VO_2_ after 2–6 months of endurance exercise training is related to increased peak exercise CO and/or A-VO_2_ Difference. When present, increased peak exercise CO is due primarily to a rise in peak exercise HR, representing a mitigation of the chronotropic incompetence typical of elderly patients, as peak stroke volume is not significantly increased after training in the majority of studies in HFREF patients (Sullivan et al., 1988; Belardinelli et al., 1996; Keteyian et al., 1996).

The intensity of the prescribed training program can also impact on rest and exercise cardiovascular function. Wisloff et al. (2007) reported that high-intensity interval aerobic training was superior to continuous moderate intensity endurance exercise training for improving resting LV ejection fraction, peripheral vascular function, and skeletal muscle mitochondrial biogenesis in elderly men with HFREF. Nechwatal et al. (2002) found that short-term high-intensity aerobic interval training significantly increased peak exercise stroke volume and CO and reduced systemic vascular resistance but there was no change in these outcomes after moderate intensity continuous endurance training in patients with HFREF.

In addition to endurance aerobic exercise training, also resistance training (RET) has shown some favorable effects in patients with HFREF, but the effect on cardiovascular performance is modest compared to that obtained with aerobic ET (Pu et al., 1997; Laoutaris et al., 2013).

## Exercise in elderly patients with heart failure and preserved LV ejection fraction (HFPEF)

HFPEF has received more attention in the recent years due to the progressive growth of the elderly population, since HFPEF patients includes a predominant percent of elderly and particularly female patients. As in HFREF patients, also HFPEF patients have a limited baseline cardiovascular performance as evaluated by peak VO_2_ values at cardiopulmonary test, and recent studies in this patient population showed that the strongest determinant of the severely reduced exercise capacity in HFPEF patients was a reduced peak A-VO_2_ Difference (Haykowsky et al., 2011).

Aerobic ET increases maximal cardiovascular performance in HFPEF patients, as reflected in an increased peak VO_2_ at cardiopulmonary stress test, but the mechanisms underlying this change are still uncertain, although an exercise training- induced increase in A-VO_2_ Difference may play an important role (Haykowsky et al., 2012). Aerobic ET may enhance A-VO_2_ Difference by improvement in peripheral vascular function leading to increase diffusive oxygen transport or by an increased oxygen utilization. Since Hundley et al. (2007) reported that resting and flow-mediated increases in leg blood flow in elderly HFPEF patients are not significantly impaired, it is possible that in this elderly population with HFPEF muscle adaptation play a more important role, compared to vascular changes, in the increase in the exercise training induced increase in A-VO_2_ Difference and peak VO_2_. In fact, Bhella et al. (2011) recently reported that elderly HFPEF patients have baseline impaired skeletal muscle oxidative metabolism, that can be favorably shifted by exercise training to a more efficient muscle O_2_ utilization. However, further studies will be required to determine the specific vascular and/or skeletal muscle mechanisms underlying the exercise training- mediated increase in peak A-VO_2_ Difference in HFPEF patients.

In addition, also central factors may play a role in the exercise training induced increase in peak VO_2_ in patients with HFPEF. Peak exercise HR is reduced in these patients (Borlaug et al., 2006), and an aerobic exercise training reverses this chronotropic incompetence (Haykowsky et al., 2012). However, the issue of an increase in CO at peak exercise after training in these patients is rather controversial and may reflect a gender difference in the response to training. In fact, in studies reporting no CO changes and rather an increase in A-VO_2_ difference the majority of HFPEF patients were women, reflecting the sex distribution of HFPEF in the general population (Haykowsky et al., 2012). Women are indeed less likely to have an exercise training induced CO or stroke volume increase, compared to men (Spina et al., 1993), and this gender difference may also translate in the HFPEF elderly population.

On the other side, there is no evidence of a beneficial effect of aerobic exercise training on the diastolic properties of LV in the HFPEF elderly population (Fujimoto et al., 2012).

## Exercise training programs

Aerobic training represents an essential component of cardiac rehabilitation programs, and had been associated to improvement in submaximal and maximal cardiovascular performance even in the elderly (Ades and Grunvald, 1990; Ades et al., 1993, 1996). Even a moderate intensity exercise training program, if followed regularly, produces favorable effects on several health outcome, if tailored on the clinical and functional state or preference of the elderly patient (Vigorito et al., 2003).

Usual exercise training programs lasting 8–12 weeks in the elderly have been associated with beneficial effects that are similar to those obtained in adults, and particularly to an increase in the cardiovascular parameters reflecting cardiovascular performance, such as maximal workload, peak VO_2_, Anaerobic Threshold, ventilatory parameters (VE/VCO_2_), or Respiratory exchange ratio, with reduction at submaximal exercise of arterial pressure and HR, indirect indicators of a reduced myocardial O_2_ consumption (Ades and Grunvald, 1990; Ades et al., 1993, 1996; Vigorito et al., 2003). Many of these studies suggest that a more extended exercise training program (>12 weeks) may be necessary to obtain beneficial effect on cardiovascular performance in elderly patients.

Training programs should include endurance aerobic exercise on a cyclette or treadmill 3 times/week. Duration of a single exercise session may vary between 5 and 60 min, according to the individual clinical and functional status and to the response to exercise, but in general is targeted to 30 min, and is inversely proportional to the intensity of exercise. Exercise session duration and intensity is gradually increased to reach the target HR, in order to control exercise induced symptoms or complications.

The training workload should be set according to the maximal predicted heart rate (MHR) or to the HR achieved at a symptom limited exercise stress test or to peak VO_2_ achieved at a cardiopulmonary stress test. Training HR should be tailored according to the functional compromise of the patient, generally ranging between 50–60% MHR (mild to moderate exercise) and 75–80% MHR (intense exercise). If HR cannot be adopted to establish the training workload, subjective perceived exertion fatigue on a 20 points Borg scale can be utilized, trying not to increase this value above 12. All sessions should be preceded by a 5–10 min of warm-up and be followed by a period of 5–10 min of recovery. In the elderly patient training is usually started at a lower workload due to the limited baseline functional capacity and to the frequently associated comorbidities negatively influencing the physical performance. In very compromised frail elderly a high intensity exercise training may induce musculo-skeletal damage or cardiovascular complications (Pollock et al., 1991).

Particularly in elderly patients with functional compromise Borg scale may be indicated to maintain the intensity of training within safe limits, provided the patients is reliable to report his symptoms during exercise. The more the elderly patients is frail or sarcopenic, the higher is the potential benefit for cardiovascular performance to include also muscle strengthening, flexibility, or balance exercise. Particularly resistance exercise, can also be useful in the elderly to improve cardiovascular performance, if adopted in conjunction with aerobic exercise training (Peterson and Gordon, 2011). Progression to high intensity exercise should be very slow or avoided in very elderly and deconditioned or frail patient, and longer warm up and recovery periods should be included into the program.

## Future perspectives and conclusions

Literature evidence indicate that aging does not represent a contraindication to exercise training in elderly healthy people and in old patients with different kind of comorbidities. Exercise programs planned for the elderly should include elements of cognitive therapy, occupational therapy and exercises designed to improve balance, all of which are directed toward enhancing the QoL of these patients. Counseling and education looking at lifestyle, dietary recommendations, medications, and self monitoring must represent all important components of this new approaching strategy to people in the more advanced age decades. Finally, larger controlled studies should be performed in order to definitively demonstrate that physical activity plays an important role to improve cardiovascular outcome in this high risk population.

### Conflict of interest statement

The Authors, Editor and Chief Editor declare that while the authors Carlo Vigorito and Francesco Giallauria as well as the editor Dario Leosco and reviewer Giovanni Esposito are currently employed by the same institution (University Federico II, Naples, Italy) there has been no conflict of interest during the review and handling of this manuscript. The authors declare that the research was conducted in the absence of any commercial or financial relationships that could be construed as a potential conflict of interest.
